# Free surface oscillation driven by rotating stirrer

**DOI:** 10.1140/epje/s10189-024-00420-z

**Published:** 2024-04-13

**Authors:** Tomoaki Watamura, Reiji Iwata, Kazuyasu Sugiyama

**Affiliations:** 1 Graduate School of Engineering, The University of Tokyo, 7-3-1, Hongo, Bunkyo-ku, Tokyo 113-8656 Japan; 2 Graduate School of Engineering Science, Osaka University, 1-3, Machikaneyama, Toyonaka, Osaka 560-8531 Japan

## Abstract

**Abstract:**

To gain insights into the mechanisms of free surface oscillation in a rotating mixing container, we observe the free surface deformation and measure the torque acting on the bar. The container was half-filled with liquids. Periodic surface oscillation occurs. At the rotational speed where the amplitude of the oscillation reaches its maximum, the time-averaged torque also takes the local maximum values. To account for the sloshing mechanism, an equation of motion is derived using the Lagrangian mechanics; we found that the sloshing occurs when the collision frequency of bar on the surface is consistent with the natural frequency of the system and the damping coefficient is sufficiently smaller than unity. The time-averaged torque increases when the sloshing becomes violent. We conclude that the hydrodynamics of oscillation is successfully modeled using point-mass mechanics, and thus we can reasonably capture the rotation speed at which violent oscillation occurs.

**Graphical Abstract:**

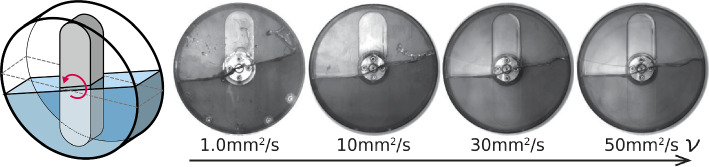
Free surface deformation driven by the rotating arm in the cylindrical container which is half-filled with liquids

## Introduction

The free surface in the moving container exhibits a drastic deformation. Several configurations are possible subjects for industrial facilities, including two immiscible fluids such as stirrer reactors [1–3], coating equipment [4–7], and food blenders [8]. Although the flow in the gap between the concentric double cylinders, i.e., a Taylor–Couette system, is a simple axisymmetric system, the gas-liquid two-phase mixture exhibits a non-axisymmetric flow pattern owing to the gravitational force [9] unless the rotor rotates around the vertical axis at relatively low rotation speed [8]. Two-immiscible fluids at rest are stratified by gravity: a light gas layer forms above, while a heavy liquid layer forms below. As a result of an advancing interface driven by fluid motion, which usually involves complex phenomena of hydrodynamic instabilities or free boundary turbulence, bubbles or droplets form therein, and strong shear also leads to the generation of an emulsion [4].

Gas-liquid two-phase flows normally appear in a vehicle power train and are related to power loss, which is categorized into windage and churning (splash) losses [10]. Windage loss is relevant to airflow and becomes significant in high-speed gears. Churning loss is related to the mixing of the gas-liquid phase in an oil-bath and accounts for most of the losses in low-speed gears [11]. Bubbles or droplets are continuously generated by the penetration of a non-axisymmetric mechanical component (e.g., crankshaft and differential gear) into the other phase. These components are partially dipped in an oil-bath; their motion causes oil splash and plays a major part in lubrication as well as by an oil-jet [12–14]. In oil-jet lubrication, the position and flow rate can be designed to maximize lubrication and heat transfer. However, dip-lubrication is a passive method; gas-liquid distribution is given by the interplay among centrifugal, gravity, viscous, and surface tension forces in fluids driven by moving bodies. In such power trains, rotating motions of a non-axisymmetric body impose periodic forcing, which causes violent waves on a gas-liquid interface [15–18]; this is called ‘sloshing’. This unsteady motion of the free surface causes mechanical damage. For this reason, the interior shape of a crankcase or oil-sump is important for suppressing sloshing and controlling the oil distribution. The fluid motion also varies the power loss during mixing; thus, for safe and efficient operations, estimating the flow pattern and controlling the flow to the desired destination a priori to its manufacture is usually required. However, because such mixing facilities consist of complex mechanical components, the details of the fluid motion and energy loss are not fully understood. Resonant free-surface flows have been the focus of extensive research on sloshing in tanks [15, 16]. The sloshing strongly interacts with the internal structures of a tank such as stiffeners, baffles, interior pipes, and pump towers. The stirrer drives liquid and also causes impulsive force when it collides with the free surface; and thus a periodic forcing is imposed on the system. During this time, the stirring body also acts as a swash bulkhead or baffle which suppresses sloshing. The orientation of stirring body varies with its rotation; therefore, the sloshing characteristics change over time. Furthermore, in mixing facilities, the bubbles modify the liquid viscosity [19] and the foam usually mitigates sloshing waves [20]. Due to these complex natures of fluid motion in mixing facilities, it is nontrivial what is an appropriate parameter to characterize the power loss in two-phase mixing.

To address the sloshing behavior which is particularly relevant to churning power loss, we introduce a simple two-phase mixing facility which allow the visual observation of the fluid motions such as generations of bubble/droplet and motion of free surface owing to the penetration of a solid into the other phase. Our objective is to derive important concepts of sloshing mechanics in a rotating mixing facility, in which a stirrer rotates around the horizontal axis. We performed experiments on the gas-liquid distribution and measured the torque acting on a rotating bar. We classified the flow patterns and found that the sloshing commences at a moderate rotation speed. The magnification of the time-averaged torque negatively correlates with the kinematic viscosity of the fluid. Suppose that a system consists of a pendulum [21, 22], the Lagrangian equations are used to investigate the motion of the free surface and torque. We successfully capture that the sloshing occurs when the frequency of bar collisions on the free surface is consistent with the natural frequency of the system and the damping coefficient is sufficiently smaller than unity.

## Experimental setup

### Experimental apparatus

**Fig. 1:**
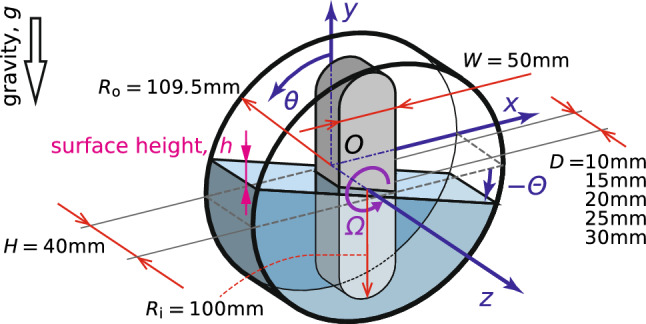
Schematic outline of the experimental configuration. The rotating bar is half immersed in working fluid: tap water or silicone oil

We conduct experiments using a mixing apparatus comprising a non-axisymmetric stirring bar, which rotates around the horizontal axis, and stationary cylindrical casing, as shown in Fig. 1. The rotating object has two stirring bars with radius $R_\text {i} = 100\,\textrm{mm}$, width $W = 50\,\textrm{mm}$, and thickness $D = 10, 15, 20, 25, 30\,\textrm{mm}$. For a typical case, we restrict ourselves to introduce the system comprising the two stirring bar. The casing with inner radius $R_\text {o} = 109.5\,\textrm{mm}$ and depth of $H = 40\,\textrm{mm}$ is made of transparent plexiglass to allow visualizing the motion of the gas-liquid interface. Tap water and silicone oil at a kinematic viscosity of $\nu = 1, 10, 30, 50\,\textrm{mm}^2/\textrm{s}$ and density of $\rho \approx 1000\,\mathrm {kg/m}^{3}$ are used as test fluids, and these temperatures are maintained at $24 \pm 3\,^\circ \textrm{C}$ which is measured by thermocouples. The kinematic viscosity of the test fluids strongly depends on temperature. From our preliminary measurements of the relationship between kinematic viscosity and temperature, the changes in the physical properties are estimated to be smaller than 9%. The physical properties of the test fluid are listed in Table 1. The casing is half-filled with the test fluid. The *x* and *y* axes are set along the horizontal and vertical directions; and the *z* axis is set along the rotating axis of the bar. Note that $\theta$ denotes the angle of the stirring bar from the vertical position; and $\varTheta$ denotes the angle of the free surface from the horizontal position. The stirring bars are driven by a speed control motor via a torque meter; its rotation speed $\varOmega$ ranges in $5\,\textrm{rpm} \le \varOmega \le 800\,\textrm{rpm}$. We run the experiments at a constant rotation speed using feedback control.

**Table 1:** Physical properties of test fluids at $25\,^\circ \textrm{C}$

	Density	Kinematic viscosity
	$\rho$ ($\mathrm {kg/m}^{3}$)	$\nu$ ($\textrm{mm}^2/\textrm{s}$)
Tap water	997	0.9
KF-96-10cs	932	10
KF-96-30cs	952	30
KF-96-50cs	957	40

### Measurement systems

**Fig. 2:**
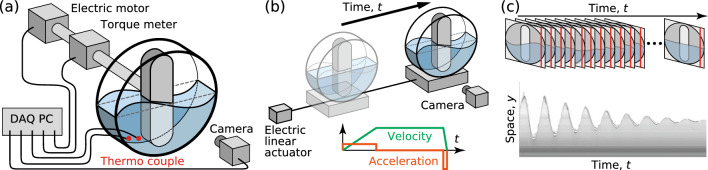
Schematic outlines of measurement setup: **a** for torque *T* and surface height *h* and **b** for dynamic characteristics in viscous-damping oscillation from *h*. **c** An example of a temporally expanded image

To investigate the effect of rotation speed on the fluid motion and torque acting on the stirring bars, we use a data acquisition computer (DAQ-PC), which allows the simultaneous measurement of temporal variations in the rotation speed $\varOmega$, torque *T*, temperature of the test fluid, and the gas-liquid distribution, as shown in Fig. 2a. The optically visualized gas-liquid distribution is captured using a high-speed video camera; in this configuration, we can visualize the gas-liquid distribution projected on the *x*–*y* plane. We conduct this simultaneous measurement over ten rotations and obtain the time-averaged value $\overline{\cdots }$, phase-averaged value $\hat{\cdots }$, and power spectrum $\tilde{\cdots }$ of the measured values. The phase average gives the ensemble average over the conditional samples at a given phase of $\Delta \theta = 2 \pi / 100$.

**Fig. 3:**
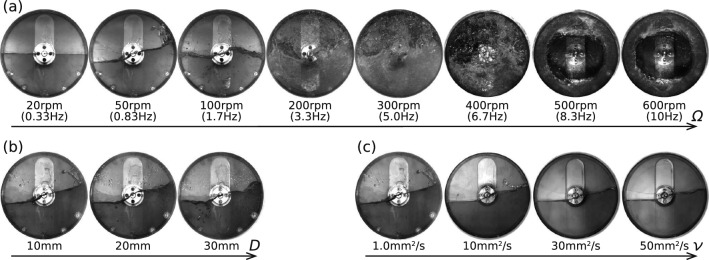
Flow pattern for **a** various rotation speeds $\varOmega$ at $D = 10\,\textrm{mm}$ and $\nu = 1.0\,\textrm{mm}^2/\textrm{s}$, **b** various bar thicknesses *D* at $\varOmega = 50\,\textrm{rpm}$ and $\nu = 1.0\,\textrm{mm}^2/\textrm{s}$, and **c** various kinematic viscosities $\nu$ at $D = 10\,\textrm{mm}$ and $\varOmega = 50\,\textrm{rpm}$

To measure the natural frequency of the liquid in the system, we also observe the impulse response of liquid motion. Fig. 2b shows a schematic outline of the impulse response measurement. The mixing apparatus is mounted on a linear electric sliding actuator, in which the stirring bar is fixed at an intended angle. The casing is initially fixed; the linear actuator translates the apparatus at a slow acceleration and stops it immediately; and the liquid sloshes due to this sudden deceleration. To probe the time response of the interface motion at a fixed position, we use the temporally expanded image extracted at the middle of the gap between the bar tip and casing $x = - (R_\text {i} + R_\text {o})/2$; a column of pixels is extracted from each frame as shown in Fig. 2c, and the gas-liquid interface is detected using the edge detection method [23]. This measurement enables us to observe the impulsive motion of the liquid in the housing and to evaluate the effect of the bar angle.

## Results and discussion

### Flow pattern

We observe the liquid phase distribution at various rotation speeds $\varOmega$, bar thickness *D*, and kinematic viscosity $\nu$ as shown in Fig. 3. Note that, on the basis of $\varOmega = 50\,\textrm{rpm}$, $D = 10\,\textrm{mm}$, and $\nu = 1.0\,\textrm{mm}^2/\textrm{s}$, one parameter is varied whereas the others are maintained constant. Firstly, from Fig. 3a, we find that the flow pattern (i.e., the distribution of the liquid phase) changes drastically and depends on the rotation speed $\varOmega$. For sufficiently low rotation speed $\varOmega \le 20\,\textrm{rpm}$, gas and liquid phases are completely stratified owing to the difference in gas-liquid densities: light gas layer forms above, while heavy liquid layer forms below; we call this flow pattern ‘stratified flow’. In this case, the free surface remains flat. For a moderate rotation speed $\varOmega > 20\,\textrm{rpm}$, the surface deformation becomes noticeable and exhibits periodic oscillation; this is called ‘sloshing flow’. At $100\,\textrm{rpm} \le \varOmega \le 400\,\textrm{rpm}$, we find that the flow includes the droplets and air slugs (bubbles); this flow pattern corresponds to the so-called ‘splash flow’. For a sufficiently high rotation speed $\varOmega \ge 500\,\textrm{rpm}$, we find a sudden transition to ‘annular (concentric air in liquid) flow’. In this case, the centrifugal force is dominant rather than the gravitational force, and thus, the liquid phase moves along the casing wall, which is known as separation by centrifugation. This flow transition occurs at the Froude number beyond unity [14] and is very similar to that observed in liquid-liquid flows [4] or foam reactors [3].

**Fig. 4:**
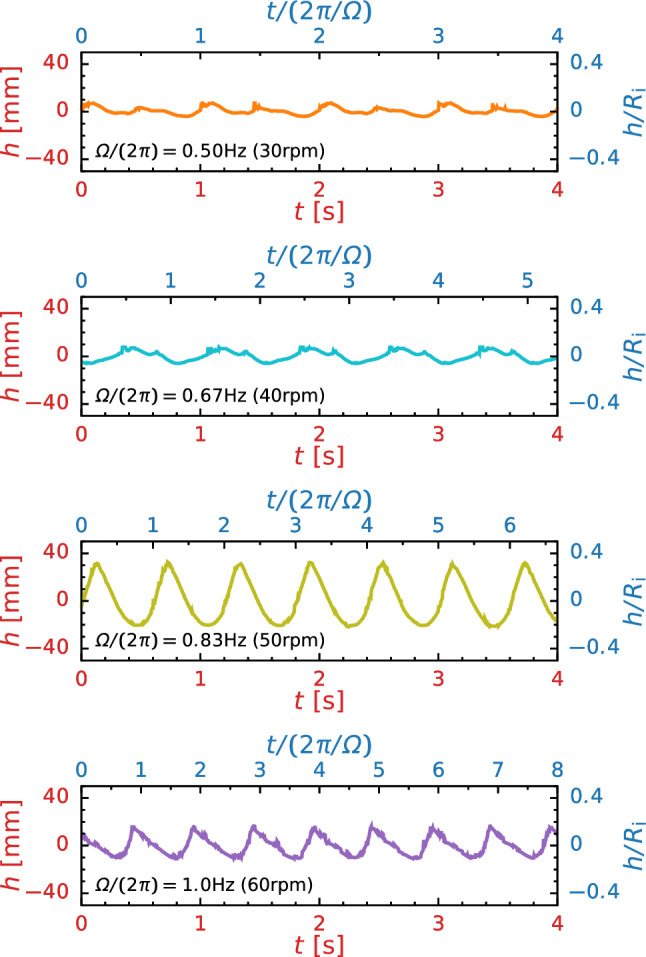
**a** Temporal variation of surface height *h* for different values of the rotation speed $\varOmega$ at $D = 10\,\textrm{mm}$ and $\nu = 1.0\,\textrm{mm}^2/\textrm{s}$

**Fig. 5:**
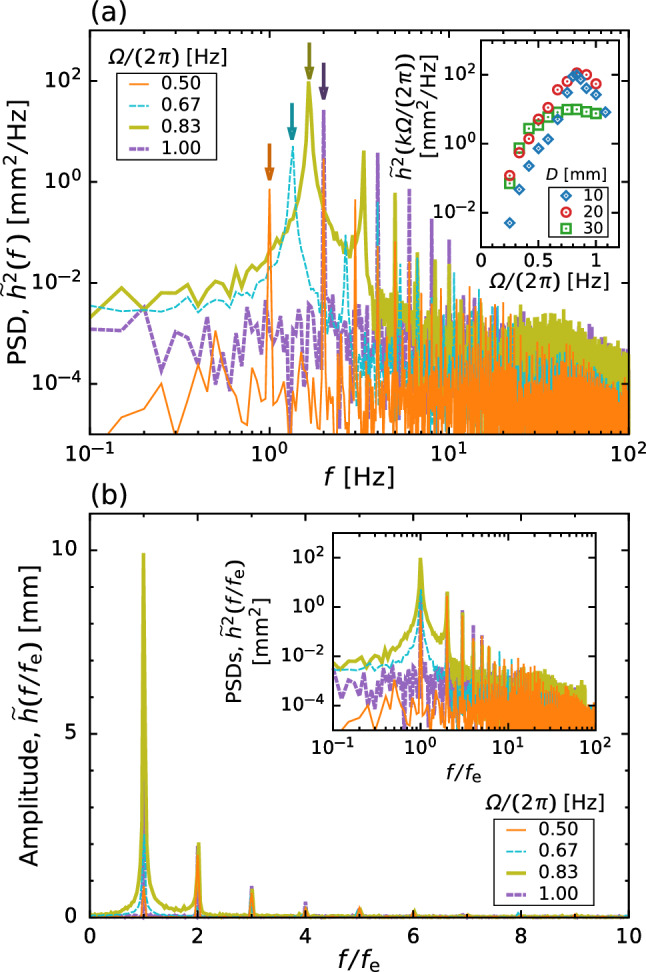
**a** Power spectrum density $\tilde{h}^2$ for different values of the rotation speed $\varOmega$ at $D = 10\,\textrm{mm}$ and $\nu = 1.0\,\textrm{mm}^2/\textrm{s}$. Arrows in (**a**) corresponds to the excitation frequency $f_\text {e} = k \varOmega /(2 \pi )$, where *k* is the number of stirring bars. The inset of (**a**) shows the response curve of liquid oscillation $\tilde{h}^2 (k \varOmega /(2 \pi ))$ vs $\varOmega /(2 \pi )$ for different *D*. **b** The amplitude $\tilde{h}$ versus normalized exciting frequency $f / f_\text {e}$. The inset shows the log-log plot of PSD $\tilde{h}^2$ versus $f / f_\text {e}$

Here, we focus on the periodic surface deformation caused by sloshing. In Fig. 3a, at $20\,\textrm{rpm} \le \varOmega \le 100\,\textrm{rpm}$, the oscillation amplitude of surface height at the casing wall increases with increasing $\varOmega$ and shows a maximum value at $\varOmega = 50\,\textrm{rpm}$, whereas it decreases in the splash flow regime ($200\,\textrm{rpm} \le \varOmega \le 400\,\textrm{rpm}$). The sloshing amplitude significantly depends on $\varOmega$ and is inferred to be maximized at a moderate $\varOmega \approx 50\,\textrm{rpm}$ (details are shown in Sect. 3.2). To overview the effect of the bar thickness *D* and viscosity of liquid $\nu$, we observe the gas-liquid distribution at $\varOmega = 50\,\textrm{rpm}$ and find that the droplets and bubbles form at small *D* (see Fig. 3b) or small $\nu$ (see Fig. 3c). Further, we find that the surface height weakly depends on *D*, but it strongly depends on $\nu$; the viscosity suppresses the sloshing amplitude. Note that we test the effect of the surfactant (Triton X-100) on the flow pattern; we observe that the addition of minute concentrations of the surfactant to tap water modifies the transition between the stratified and annular flow regimes modified but has only a minor effect on the sloshing behavior. The addition of the surfactant reduces the surface tension [24] and prevents bubble coalescence due to the Marangoni effect [25]. The bubble suspension behaves as a non-Newtonian liquid, leading to modifications turbulence and momentum transfer. For this reason, the addition of surfactant significantly affects the flow transition, similar to that in food blenders [8]; however, the detail discussions are beyond the present scope.

**Fig. 6:**
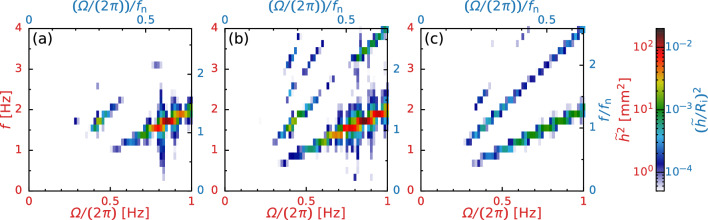
Power spectrum map for various rotation speeds $\varOmega$ at $\nu = 1.0\,\textrm{mm}^2/\textrm{s}$: **a** $D = 10\,\textrm{mm}$, **b** $D = 20\,\textrm{mm}$, and **c** $D = 30\,\textrm{mm}$

**Fig. 7:**
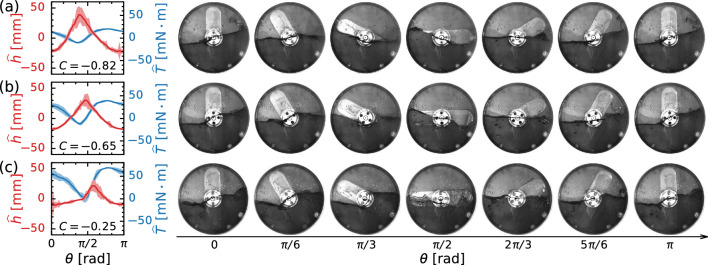
Phase averaged values of surface height $\hat{h}$ and torque $\hat{T}$ (Left), and typical snapshots of liquid distributions (Right) at $\varOmega = 0.83\,\textrm{Hz}$ (50 rpm) and $\nu = 1.0\,\textrm{mm}^2/\textrm{s}$: **a** $D = 10\,\textrm{mm}$, **b** $D = 20\,\textrm{mm}$, and **c** $D = 30\,\textrm{mm}$. The shaded regions in the left panel correspond to envelopes given by $\pm 3$ standard deviation over 10 rotations

### Sloshing amplitude

We now investigate the temporal variation of the surface height *h* to evaluate the amplitude-frequency characteristics of sloshing. Note that we measure the surface height *h* at $x = -(R_\text {i} + R_\text {o})/2$, at which we can measure the maximum surface elevation due to the collision of stirring bar on free surface. Figure 4a shows the temporal variation in the surface height at the bar thickness of $D = 10\,\textrm{mm}$ and the kinematic viscosity of $\nu = 1.0\,\textrm{mm}^2/\textrm{s}$ for various rotation speeds $\varOmega$. Although entrained bubbles and the meniscus of moving contact lines cause irregular ripples on the free surface, they have only a minor effect on the measurement because the ripple heights are fairly small compared to the sloshing motion. The surface height *h* is found to exhibit repeatable wave motion and has the maximum amplitude at $\varOmega \approx 50\,\textrm{rpm}$. For a more quantitative evaluation, Fig. 5a shows the power spectrum density (PSD) of the surface height $\tilde{h}^2$. The arrows indicate the excitation frequency of the rotating stirring bar $f_\text {e} = k \varOmega /(2 \pi )$, where *k* is the number of stirring bars. Figure 5b shows the relationship between the amplitude of surface oscillation $\tilde{h}$ and scaled frequency $f / f_\text {e}$. From Fig. 5a, b, we can find that $\tilde{h}$ takes the maximum value at the excitation frequency $f_\text {e}$ in most cases. However, for $\varOmega = 30\,\textrm{rpm}$, $\tilde{h}$ takes the maximum value at the second harmonic frequency $2f_\text {e}$ (see Fig. 5b), implying that the locked-in oscillation appears at $f \approx 2\,\textrm{Hz}$. We can also find that the prominent peak is remarkably sharp and high-order harmonics are noticeable in Fig. 5b. These results indicate the formation of sawtooth wave due to the nonlinear response of free surface motion to the collision of stirring bar on free surface; this waveform can be seen from Fig. 4. The inset of Fig. 5a shows the frequency response curve (i.e. the relationship between $\tilde{h}^2 (k \varOmega /(2 \pi ))$ and $\varOmega /(2 \pi )$) for various *D*. We find $\tilde{h}^2$ takes the maximum value at $f \approx 0.8\,\textrm{Hz} \; (= 48\,\textrm{rpm})$ which is nearly independent of *D*, while the tail-decay behavior is highly correlated with *D*. Further, to outline the amplitude-frequency characteristics of sloshing, we draw a colormap of $\tilde{h}^2$ in the *f*–$\varOmega$ plane for various *D* as shown in Fig. 6. This colormap displays that the oscillation of the sloshing wave consists of a fundamental wave $f = k \varOmega /(2 \pi )$ and also its harmonics; this feature is independent of $\varOmega$ and *D*. We also find that the amplitude of surface oscillation takes the maximum value at $f \approx 1.8 \,\textrm{Hz}$, which is likely to be involved with the natural frequency of this system $f_\text {n}$ (details are discussed in Sect. 3.4).

**Fig. 8:**
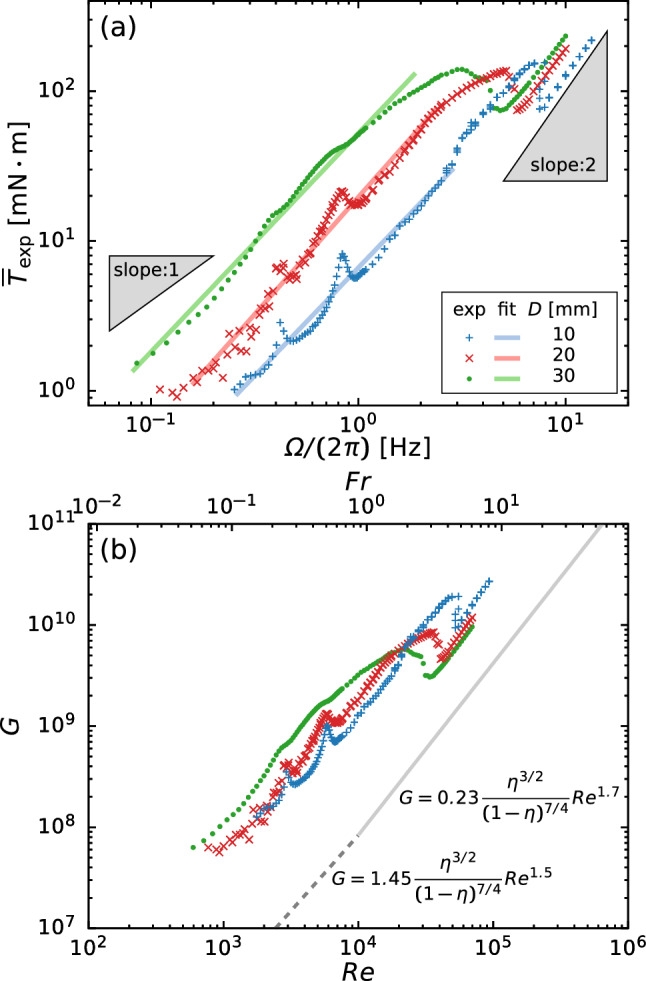
**a** Time-averaged torque $\overline{T}_\text {exp}$ versus rotation speed $\varOmega$. Fitted lines are obtained through least mean square method. Triangles are a guides to eye of the slope; $\overline{T}_\text {exp} \propto 1  \&  2$. **b** Dimensionless torque *G* versus the Reynolds number ${\textit{Re}}$ and the Froude number *Fr*. The lines show Wendt’s empirical relation for Taylor–Couette flows [26]

### Torque behavior

The distribution and motion of the liquid are reflected in the torque acting on the stirring bar. Figure 7 depicts the phase-averaged values of the surface height $\hat{h}$ and torque $\hat{T}$ over ten rotations, which are displayed together with the temporal variation of the liquid distribution. The deviation of the surface height increases at $\theta = 0$ and $\pi /2$ owing to the formation of bubbles, droplets, and the meniscus of the moving contact line. $\hat{h}$ and $\hat{T}$ show highly reproducible wave motions, and are negatively correlated with the correlation coefficient $C (= \text {cov} ( \hat{h}, \hat{T})/[\sigma (\hat{h}) \sigma (\hat{T})])$ of $-0.82 \le C \le -0.25$, where cov is the covariance and $\sigma$ is the standard deviation. From visual observation, we find that a traveling wave is imposed on the free surface for a large *D* (i.e., thicker stirring bar); for this reason, $\hat{h}$ and $\hat{T}$ show considerable phase differences, which results in a decrease in the magnitude of *C*.

The time-averaged torque is crucial for designing a mechanical system a priori to its operation. Figure 8a depicts the experimentally obtained time-averaged torque $\overline{T}_\text {exp}$ as a function of rotation speed $\varOmega$, which includes three remarkable aspects. Firstly, $\overline{T}_\text {exp}$ shows a local maximum at $\varOmega /(2 \pi ) \approx 5\,\textrm{Hz}$, at which the transition from stratified to annular flow occurs (see Sect. 3.1 and Fig. 3a). Secondly, the scaling exponent (i.e., the slope *s* in the power-law scaling of torque $\overline{T}_\text {exp} \sim \varOmega ^s$) depends on the flow pattern: $s \approx 1.5$ for stratified flow and $s \approx 2$ for annular flow; the details are summarized in Table 2. This relation is analogous to the torque scaling in Taylor–Couette flows: $s \approx 1.5$ for the transient regime and $s \approx 2$ for the fully turbulent regime of [26–29]. Note that we use a least-square fitting that models the torque as $\overline{T}_\text {fit} = a \varOmega ^s$ and obtain *s* for $\varOmega < \varOmega _\text {M}/2$ (stratified flow) and $\varOmega > 1.5 \times \varOmega _\text {M}$ (annular flow), where $\varOmega _\text {M}$ is the position at which $\overline{T}_\text {exp}$ shows the local maximum at $\varOmega \approx 5\,\textrm{Hz}$. In the transient regime of a Taylor–Coutte flow in which the inner cylinder rotates whereas the outer one is stationary, the nondimensional torque *G* is uniquely scaled as [26]

**Table 2:** Comparison of the slope *s* in the torque scaling $\overline{T}_\text {fit} = a \varOmega ^s$

$D (\textrm{mm})$	Stratified flow regime	Annular flow regime
	$\varOmega < \varOmega _\text {M}/2$	$\varOmega > 1.5 \times \varOmega _\text {M}$
10	$1.44 \pm 0.03$	$1.90 \pm 0.02$
20	$1.56 \pm 0.02$	$1.84 \pm 0.01$
30	$1.45 \pm 0.02$	$1.81 \pm 0.01$

**Table 3:** Nondimensional numbers

Description	Symbol	Value	Related study on two-phase flow
Reynolds number	${\textit{Re}} = \varOmega R_\text {i} (R_\text {o} - R_\text {i}) / \nu$	$\mathcal {O}(10^{1})$–$\mathcal {O}(10^{5})$	[9, 12, 13]
Froude number	${\textit{Fr}} = \varOmega \sqrt{ R_\text {i} / g}$	$\mathcal {O}(10^{-1})$–$\mathcal {O}(10^{1})$	[8, 12, 14]
Aspect ratio of casing	$\alpha = H/R_\text {o}$	0.46	
Aspect ratio of stirring bar	$\beta = W/R_\text {i}$	0.50	
Thickness ratio	$\gamma = D/H$	0.25–0.75	
Radius ratio	$\eta = R_\text {i}/R_\text {o}$	0.91	

1 $$\begin{aligned}&G \left( \equiv \frac{T}{\rho \nu ^2 D} \right) \nonumber \\&\quad = \left\{ \begin{array}{l} 1.45 \dfrac{\eta ^{3/2}}{(1-\eta )^{7/4}}{\textit{Re}}^{1.5} \\ \qquad \qquad (4 \times 10^2< Re< 10^4) \\ 0.23 \dfrac{\eta ^{3/2}}{(1-\eta )^{7/4}}Re^{1.7} \\ \qquad \qquad (10^4< Re < 10^5) \end{array} \right. , \end{aligned}$$

where $\eta =R_\text {i}/R_\text {o}$ is the radius ratio and *Re* is the Reynolds number given by

2 $$\begin{aligned} {\textit{Re}} = \frac{\varOmega R_\text {i} (R_\text {o} - R_\text {i})}{\nu }. \end{aligned}$$

The relationship between *G* and ${\textit{Re}}$ is shown in Fig. 8b, in which the Froude number is also indicated. Although *G* should be insensitive to the thickness of the stirring bar *D* in this empirical expression Eq. (1), *G* depends strongly on *D* in any flow regime. Furthermore, the transition to annular flow also depends strongly on *D*; the onset ranges broadly within $2 \le {\textit{Fr}} \le 5$, where ${\textit{Fr}}$ is the Froude number given as

3 $$\begin{aligned} {\textit{Fr}} = \varOmega \sqrt{\frac{R_\text {i}}{g}}. \end{aligned}$$

Although there is a clear inconsistency in the magnitude of the nondimensional torque due to the shape difference, the scaling exponent of torque is globally similar to the one in Taylor–Couette flows. However, a detailed and accepted explanation of the torque behavior is lacking due to the complex phenomena of two-phase flow. We now restrict ourselves to study on the sloshing at $\varOmega /(2 \pi ) \approx 0.8$ Hz because violent sloshing often causes mechanical damage on a system. Thirdly, in the stratified flow regime, $\overline{T}_\text {exp}$ also exhibits steep local maxima at $\varOmega /(2 \pi ) \approx 0.4$ and 0.8 Hz. The latter rotation speed ($\varOmega /(2 \pi ) \approx 0.8\,\textrm{Hz}$) is consistent with that at which $\tilde{h}^2$ reaches its maximum value (see the inset of Figs. 5a and 6), leading to that the increase of churning loss is particularly relevant to the fluid motion, and vice versa. Here, we define the magnification factor $\overline{T}_\text {exp}/\overline{T}_\text {fit}$, which is the ratio of the experimentally measured time-averaged torque $\overline{T}_\text {exp}$ to the empirically fitted value $\overline{T}_\text {fit}$.

**Fig. 9:**
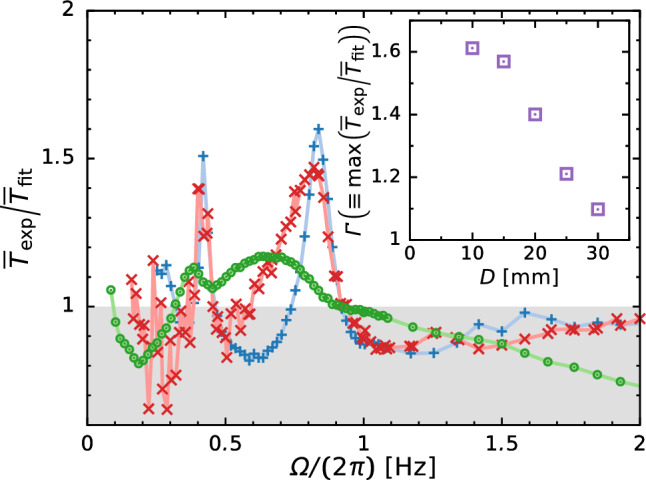
Magnification factor of time-averaged torque versus $\varOmega$. Inset shows the maximum magnification factor of time-averaged torque $\varGamma$ for different values of bar thickness *D*

Figure 9 shows the magnification factor as a function of $\varOmega$. $\overline{T}_\text {exp}/\overline{T}_\text {fit}$ strongly depends on *D*. The maximum magnification factor $\varGamma ( \equiv \max (\overline{T}_\text {exp}/\overline{T}_\text {fit}))$ shows a maximum value of $\varGamma = 1.6$ at $D = 10$ mm and decreases with increasing *D*. This is because increasing the bar thickness reduces the axial gap $\delta (\equiv H - D)$; thus, the viscous force $\sim \rho \nu \varOmega R_\text {i}/\delta$ suppresses the fluid motion. For this reason, the sloshing amplitude $\tilde{h}$ decreases and exhibits a long-tail distribution in its decay behavior (see inset of Fig. 5a). In such a dissipative system, the energy input from external forcing is required to maintain the sloshing motion, that is to increase the potential energy. Therefore, the time-averaged torque $\overline{T}$ (= (time-averaged energy input) $\div$ (angular velocity)) increases when the sloshing motion becomes violent.

### Modeling of oscillation dynamics

As shown in Sects. 3.2 and 3.3, we have observed the complex two-phase flow phenomena leading to violent sloshing. The maximization of the torque is well correlated with the sloshing, which is quantitatively characterized by the surface height. In this section, based on analytical mechanics, we present the simplest model of the inertial and potential forces of the stirring bar and the liquid phase, under the very bold but physically consistent assumption that the displacement of the free surface can be described only by a single oscillation mode in which the free surface remains planar. From this assumption, a simple pendulum model is derived and is extended to include dissipation mechanisms and external forcing. Herein, we demonstrate that the maximization of torque identified in the previous section can be reasonably explained by our pendulum model. The proposed model, despite its oversimplification, can capture the essence of the violent sloshing phenomenon that leads to the maximization of torque.

Here, we introduce several nondimensional numbers based on the Buckingham theorem [30]. We consider the relevant values: the radius of bar and casing $R_\text {i}, R_\text {o}$; the width of bar *W*; the thickness of bar and casing *D*, *H*; the density and viscosity of liquid $\rho , \mu$; the rotation speed $\varOmega$; and the acceleration of gravity *g*. In the present gas-liquid two-phase system, the density and viscosity of air are sufficiently small. From our preliminary experiment, we confirm that the sloshing behavior is insensitive to the variation of surface tension. For these reasons, we neglect the dynamics of flow in gas-phase and the interracial phenomena including wetting properties. We now obtain 6 nondimensional parameters: the Reynolds number ${\textit{Re}}$; the Froude number ${\textit{Fr}}$; the aspect ratio of casing $\alpha$ and stirring bar $\beta$; the thickness ratio $\gamma$; and the radius ratio $\eta$, as summarized in Table 3. The motion of the fluid is nondimensionalized as follows:

4 $$\begin{aligned} \frac{D \varvec{u}}{Dt} = -\varvec{\nabla } p +\frac{1}{{\textit{Re}}}\varvec{\nabla }^2 \varvec{u} +\frac{1-\eta }{\eta }\frac{1}{{\textit{Fr}}^2}\varvec{e}_y +\varvec{f}_\text {ext}, \end{aligned}$$

where $\varvec{f}_\text {ext}$ is external forcing by the stirring bar which varies in space and time. Although we can estimate the torque acting on the rotating stirrer by using DNS, the computational cost of DNS does not allow us to measure the torque in parametric way to resolve the local maxima in *T*–$\varOmega$ curve. Nevertheless, for hypothetical estimation of the onset of violent sloshing, a priori experiment or simulation is important rather than an accurate simulation. We then focus on revealing what is an appropriate variable in the onset and violence of sloshing.

**Fig. 10:**
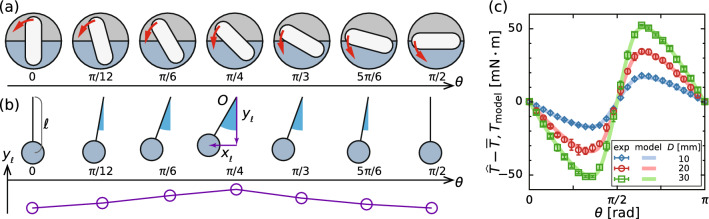
Schematic of the pendulum model. Displacement of **a** fluid volume and **b** center of gravity of the pendulum. **c** Relationship between the torque and the position of rotating bar at $\varOmega = 0.1\,\textrm{Hz}$ (6 rpm). Error bars show standard deviation over 20 rotations

To puzzle the sloshing phenomena, at first, we shall model the liquid distribution and the torque acting on the stirring bar using the simplest linearized motion of the free surface [15, 21, 22], in which the free surface remains planar (as outlined in Fig. 1). Figure 10a illustrates the displacement of fluid volume by the rotating stirring bar and Fig. 10b outlines an equivalent pendulum describing the movement of its center of gravity, where $\ell$ represents the length of the pendulum. For the point-mass modeling, we consider the liquid domain which subtracts the half-immersed stirring bar from the half-cylinder. Based on hydrostatics, we consider the rotational energy of the stirring bar $K_\text {B} (= I_\text {B} \dot{\theta }^2/2)$, kinetic energy of liquid $K_\text {L} (= I_\text {L} \dot{\varTheta }^2/2)$, and potential energy of liquid $\varPi _\text {L}$. $I_\text {B}$ and $I_\text {L}$ are the moments of inertia of the stirring bar and the liquid as follows:

5a $$\begin{aligned} \frac{I_\text {B}}{\rho _\text {B}}&= \underbrace{ 8\int _0^{W/2} \int _0^{R_\text {i}-W/2} \int _0^{D/2} (x^2+y^2) \, \text {d}x \text {d}y \text {d}z }_{\text {cuboid}} + \underbrace{ 8\int _0^{W/2} \int _0^{\pi /2} \int _0^{D/2} \left\{ (r \cos \theta )^2+(r \sin \theta +R_\text {i} - W/2)^2\right\} r \, \text {d}r \text {d}\theta \text {d}z }_{\text {rounded tip}} \nonumber \\&= \left\{ \frac{W(2 R_\text {i} - W)(R_\text {i}^2-R_\text {i}W+W^2)}{3} + \frac{\pi W^2 (8 R_\text {i}^2-8 R_\text {i}W+3 W^2)}{32} \right\} D, \end{aligned}$$

5b $$\begin{aligned} \frac{I_\text {L}}{\rho }&= \underbrace{ \int _0^{R_\text {o}} \int _0^{\pi /2} \int _0^{H} r^3 \text {d}r \text {d}\theta \text {d}z }_{\text {half-cylinder}} - \underbrace{ \frac{I_\text {B}}{2\rho _\text {B}} }_{\text {stirring bar}}\nonumber \\&= \frac{\pi R_\text {o}^4 H}{4}- \left\{ \frac{W(2 R_\text {i} - W)(R_\text {i}^2-R_\text {i}W+W^2)}{6} + \frac{\pi W^2 (8 R_\text {i}^2-8 R_\text {i}W+3 W^2)}{64} \right\} D, \end{aligned}$$

5c $$\begin{aligned} \frac{\varPi _\text {L}}{\rho g}&= \underbrace{ \int _0^{R_\text {o}} \int _{\pi +\varTheta }^{2\pi +\varTheta } \int _0^{H} r^2 \sin \theta \, \text {d}r \text {d}\theta \text {d}z }_{\text {half-cylinder}} - \underbrace{ \frac{V_\text {B}}{2} y_\text {c} (\theta ,\varTheta ) }_{\text {stirring bar}}= - \frac{2 R_\text {o}^3 H}{3} \cos \varTheta - \frac{V_\text {B}}{2} y_\text {c} (\theta ,\varTheta ), \end{aligned}$$

5d $$\begin{aligned} V_\text {B}&= \underbrace{ W (2R_\text {i} - W)D }_{\text {cuboid}} + \underbrace{ \frac{\pi W^2 D}{4} }_{\text {rounded tip}}, \end{aligned}$$

where $\theta$ and $\varTheta$ are the angles of the stirring bar and free-surface (see Fig. 1), *g* is the acceleration of gravity, $\rho _\text {B}$ is the density of stirring bar, $V_\text {B}$ is the volume of the stirring bar, and $y_\text {c}$ is the *y*-component of the center of gravity of the stirring bar which is half-immersed below the free surface (details are given in Appendix A). The Lagrangian for this system *L* can be defined as follows:

6 $$\begin{aligned} L{} & {} =K_\text {B}+K_\text {L}-\varPi _\text {L}\nonumber \\{} & {} = \frac{I_\text {B}}{2} \dot{\theta }^2 +\frac{I_\text {L}}{2} \dot{\varTheta }^2 +\frac{2 \rho g R_\text {o}^3 H}{3} \cos \varTheta \nonumber \\{} & {} \quad +\frac{\rho g V_B}{2} y_\text {c} (\theta ,\varTheta ), \end{aligned}$$

Using the Lagrange equation with imposing an external moment (i.e., torque), the equation for rotational motion can be written as follows:

7 $$\begin{aligned} \frac{\textrm{d}}{\textrm{d}t} \left( \frac{\partial L}{\partial \dot{\theta }} \right) - \frac{\partial L}{\partial \theta }{} & {} = I_\text {B} \ddot{\theta } -\frac{\rho g V_\text {B}}{2} \frac{\partial y_\text {c}}{\partial \theta }\nonumber \\{} & {} = T, \end{aligned}$$

where

8 $$\begin{aligned} \frac{\partial y_\text {c}}{\partial \theta }&= - \sum _{n=1}^{\infty } 4 n^2 C_{2n} \cos \left\{ 2n (\theta -\varTheta ) \right\} \sin \varTheta \ \nonumber \\&\quad - 2 \sum _{n=1}^{\infty } n C_{2n} \sin \left\{ 2n (\theta -\varTheta ) \right\} \cos \varTheta . \end{aligned}$$

Note that the stirring bar rotates at a constant rotation speed $\dot{\theta } (= \varOmega )$; hence, its acceleration vanishes (i.e., $\ddot{\theta } = 0$). Here, we consider a very slow rotation speed, in which the surface height is negligibly small (i.e., $\varTheta \rightarrow 0$). From Eqs. (6) and (7), the torque acting on the stirring bar can be modeled as follows:

9 $$\begin{aligned} T_\text {model} = \rho g V_\text {B} \sum _{n=1}^{\infty } n C_{2n} \sin \left( 2n \theta \right) . \end{aligned}$$

A comparison between the modeled torque $T_\text {model}$ (Eq. (8)) and the experimentally obtained phase-averaged torque which subtracted time-averaged torque $\hat{T}-\overline{T}$ is shown in Fig. 10c, where the rotation speed is very low $\varOmega /(2 \pi ) = 0.1\,\textrm{Hz}$ in the experiments; the results show excellent agreement between $T_\text {model}$ and $\hat{T} - \overline{T}$. Consequently, when the free surface remains stationary, the torque acting on the stirring bar can be reasonably predicted from the distribution of the hydrostatic pressure around it, i.e., the buoyancy. At high rotation speed, the time-averaged torque becomes larger than the fluctuation component and can be predicted using empirical relation in Table 2, even though the model equation presented in Eq. (9) cannot estimate the universal fluctuation component.

Next, the motion of the liquid is given as follows:

10 $$\begin{aligned} \frac{\textrm{d}}{\textrm{d} t} \left( \frac{\partial L}{\partial \dot{\varTheta }} \right) - \left( \frac{\partial L}{\partial \varTheta } \right)&= I_\text {L} \ddot{\varTheta } + \frac{2 \rho g R_\text {o}^3 H}{3} \sin \varTheta \ \nonumber \\&\quad - \frac{\rho g V_\text {B}}{2} \frac{\partial y_\text {c}}{\partial \varTheta } \nonumber \\&= -T, \end{aligned}$$

where

11 $$\begin{aligned} \frac{\partial y_\text {c}}{\partial \varTheta }&= \Bigg [ \sum _{n=1}^{\infty } (4n^2-1) C_{2n} \nonumber \\&\quad \times \cos \left\{ 2n (\theta -\varTheta ) \right\} -C_0 \Bigg ] \sin \varTheta . \end{aligned}$$

Suppose that the surface deformation is small $|\varTheta | \ll 1$. By adding a viscous term into Eq. (9) and linearizing it with respect to $\varTheta$, we obtain the equation for a damped harmonic oscillator as follows:

12 $$\begin{aligned}&\ddot{\varTheta } + 2 \zeta \omega _\text {n} \dot{\varTheta } + \omega _\text {n}^2 \varTheta = \phi ,\nonumber \\&\quad \left\{ \begin{array}{l} \phi = -I_\text {L}^{-1}T, \\ \omega _\text {n} = \sqrt{\dfrac{m g \ell }{I_\text {L}}}, \\ \zeta = \dfrac{c}{\sqrt{m g \ell I_\text {L}}} = \dfrac{1}{\omega _\text {n} \tau _\text {d}}, \displaystyle \end{array} \right. \end{aligned}$$

where $\omega _\text {n} (\equiv 2 \pi f_\text {n})$ is the natural angular frequency, $f_\text {n}$ is the natural frequency, *c* is the damping coefficient, and $\zeta$ is the damping ratio, $\tau _\text {d}$ is the damping period, $m (= \rho (\pi R_\text {o}^2H/2 - V_\text {B}/2))$ is the mass of fluid, $\ell$ is the length of the pendulum (see Fig. 10b), i.e., the distance between the center of system *O* to the center of mass of the liquid given by

13 $$\begin{aligned} \ell&= \frac{\rho }{m} \Bigg \langle \frac{2 R_\text {o}^3 H}{3}\end{aligned}$$

14 $$\begin{aligned}&\quad -\frac{V_\text {B}}{2} \Bigg \{ C_0 - \sum _{n=1}^{\infty } (4n^2-1) C_{2n} \cos \left( 2n \theta \right) \Bigg \} \Bigg \rangle _\theta \nonumber \\&= \frac{\rho }{m} \left( \frac{2 R_\text {o}^3 H}{3} -\frac{V_\text {B}}{2} C_0 \right) , \end{aligned}$$

where $\langle \cdots \rangle _\theta$ stands for the average in the range of $\theta \in [0,2 \pi ]$. Here, we extend a mechanical pendulum model in which the fluid is considered as a solid half-cylinder [21, 22].

**Fig. 11:**
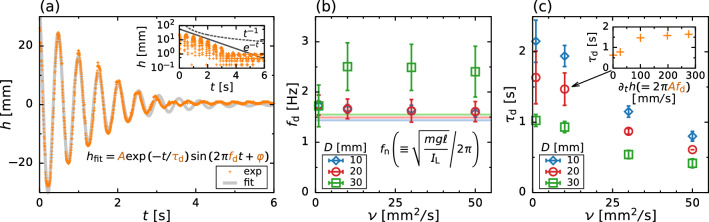
**a** Viscous-damping of surface height. The inset shows log-normal plot to clarify the decay of *h*. **b** Oscillation frequency $f_\text {d}$ and **c** damping period $\tau _\text {d}$ versus kinematic viscosity $\nu$ for different bar thickness *D*. In (**b**), $f_\text {n}$ denotes the natural frequency of the system. Error bars show the minimum and maximum values in several experimental runs. The inset of (**c**) shows the relationship between $\tau _\text {d}$ and velocity of surface oscillation $\partial h / \partial t$, where we plot several instances every 1 s of time-sequential data shown in (**a**)

**Fig. 12:**
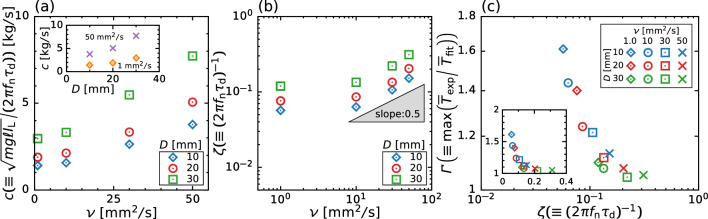
**a** Damping coefficient *c* and **b** damping ratio versus kinematic viscosity $\nu$ for different bar thickness *D*. The inset of (**a**) shows the relationship between *c* and *D* at $\nu = 1  \&  50\,\textrm{mm}^2/\textrm{s}$. **c** Log–log plot of the maximum magnification factor of time-averaged torque $\varGamma$ versus damping ratio $\zeta$. The inset shows the linear–linear plot to clarify the decay of $\varGamma$ to unity for $\zeta \rightarrow 1$

To confirm the validity of this modeling and to evaluate the damping coefficient *c*, we observe the impulsive response of the damped oscillation of the free surface (see Fig. 2b). Figure 11a shows the temporal variation in the surface height *h* after imposing an impulsive force. We estimate the damping frequency $f_\text {d}$ and damping period $\tau _\text {d}$ by using the least squares method, in which the fitting model is given as $h_\text {fit} = A \exp (-t/\tau _\text {d}) \sin (2 \pi f_\text {d}+\varphi )$. We find that the model differs only slightly from the experimental data at $t > rsim 3 \text {s}$. From Fig. 8 and Table 2, we can find that the torque resistance is proportional to $\approx \varOmega ^{1.5}$ owing to the nonlinearity of flow, in which the drag force becomes to proportional to the square of velocity. Therefore, the oscillation of surface height *h* shows not only an exponential decay (viscous damping) but also a reciprocal decay (quadratic-velocity damping) [31]. The inset of Fig. 11a shows the decay of *h* with respect to *t*, indicating excellent correlation with $e^{-t}$ at $t \lesssim 3\,\text {s}$ and $t^{-1}$ at $t > rsim 3\,\text {s}$. For this reason, the model shows noticeable difference from the experimental data at $t > rsim 3\,\text {s}$. whereas at the initial stage of the surface oscillatio $t \lesssim 3\,\text {s}$, $h_\text {fit}$ is found to be in good agreement with $h_\text {exp}$; Therefore, this result confirms that the proposed model can reasonably captures the damped harmonic oscillation. The dependence of $f_\text {d}$ and $\tau _\text {d}$ on the kinematic viscosity of liquids $\nu$ are respectively shown in Fig. 11b, c. The horizontal lines in Fig. 11b indicate the natural frequency $f_\text {n} (\equiv \omega _\text {n}/(2 \pi ))$ given by Eqs. (12) and (13). In this configuration, we can see that $f_\text {n}$ depends only weakly on the bar thickness *D*. The experimental results are fully consistent with the modeled ones, even though there is a clear difference between the modeled and experimental results at $D = 30\,\textrm{mm}$ and $\nu \ge 10\,\textrm{mm}^2/\textrm{s}$; the frequency $f_\text {d}$ depends strongly on the angle of the stirring bar. For small $\nu$, the values $f_\text {n}$ and $f_\text {d}$ are of the same order; therefore, we can confirm that the pendulum model [21, 22] reasonably simulates that violent sloshing commences at the resonance frequency of $f_\text {n} (\equiv \omega _\text {n}/(2 \pi ))$. The damping period $\tau _\text {d}$ also characterizes the response of the fluid motion. As shown in Fig. 11c, $\tau _\text {d}$ monotonically decreases as a function of $\nu$ because the viscosity of the liquid involves damping of its motion. Note that the drag force viscous and pressure drag reduce the amplitude of surface oscillation; the former is proportional to velocity and the latter is proportional to square of one. From Fig. 11a, we analyze the time dependence of damping period $\tau _\text {d}$ and then obtain the relationship between $\tau _\text {d}$ and the velocity $\partial h / \partial t = 2 \pi A f_\text {d}$ as shown in the inset of Fig. 11c. We find that $\tau _\text {d}$ is insensitive to $\partial h / \partial t$, except at low-velocity region $\partial h / \partial t \lesssim \mathcal {O}(10)$ mm/s. From Fig. 4, in the operation condition at which violent sloshing occurs, we find that the maximum amplitude shows $h \approx 40$ mm and surface velocity takes $\partial h / \partial t \approx 400$ mm/s. In this case, $\tau _\text {d}$ is independent of the velocity of surface oscillation. Moreover, the stirring bar in the present configuration has sharp corners; the separation point is fixed (i.e., not Reynolds number dependent). From our preliminary experiments on the impact of size effect on sloshing motion, we confirm that the effect of the Reynolds number on the damping behavior is less significant [16].

To further analyze the damping behavior of surface oscillation, we plot the dependence of the damping coefficient *c* and damping ratio $\zeta$ on the kinematic viscosity $\nu$ respectively in Fig. 12a, b. Note that we evaluate *c* and $\zeta$ from the experimentally measured value $\tau _\text {d}$, and thus these values include $\pm 20$% uncertainty at the maximum arising from broad deviation of experimental duplicates. From these figures, we can observe *c* and $\zeta$ are correlated with $\nu$ but depends strongly on *D*, indicating that the power-law relation in $\zeta$–$\nu$ with exponent $\approx 0.5$. The scaling behavior is in excellent accord with the empirical relation [15, 32]. Following the previous study on the viscous damping of sloshing, the magnitude of $\zeta$ depends on the tank geometry, i.e., shape and liquid level; and thus we can evaluate how sloshing is violent by measuring the damping characteristics and estimate the viscosity at which sloshing occurs. Figure 12c shows the relationship between the maximum magnification factor $\varGamma (\equiv \max (\overline{T}_\text {exp}/\overline{T}_\text {fit}))$ and damping coefficient $\zeta$. For small $\zeta$, $\varGamma$ negatively correlates with the $\zeta$ and is insensitive to the thickness of the stirring bar *D*. In this region, the effect of viscosity becomes less prominent; thus, the sloshing commences and its amplitude negatively correlates with the kinematic viscosity $\nu$ (see Fig. 11c). Although the viscous force has a minor effect on sloshing, viscous dissipation reduces the energy of fluid motion; violent sloshing requires extra energy input to maintain its amplitude. Qualitatively, the increased $\varGamma$ at small $\zeta$ is attributed to the energy balance with the magnitude of the surface height, even though we cannot explain the unique scaling relation between $\varGamma$ and $\zeta$. For a sufficiently large $\zeta$, $\varGamma$ weakly depends on $\zeta$ and saturates at unity, indicating that the surface height is likely to become small in a high-viscosity liquid; as shown in Fig. 8, the local maxima (i.e., the extra energy input) no longer appear in the *T*–$\varOmega$ curve.

**Fig. 13:**
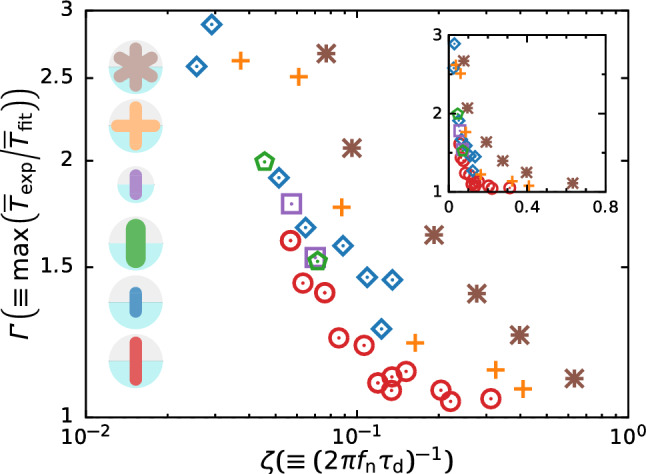
Same as Fig. 12c but for different shapes of stirring bars: standard bar (red), shorter bar (blue), wider bar (green), smaller system (purple), four-bar (orange), and six-bar (brown). The inset shows the linear-linear plot to clarify the decay of $\varGamma$ to unity for $\zeta \rightarrow 1$

We repeat the experiments on the different shapes of the stirring bar and find similar flow patterns, i.e., stratified, sloshing, splashing, and annular flow modes, regardless of the shape of the stirring bar. The relationship between $\varGamma$ and $\zeta$ for the various bars is shown in Fig. 13. We find striking features: a negative correlation between $\varGamma$ and $\zeta$ and saturation of $\varGamma$ at unity, even though $\varGamma$–$\zeta$ curves depend on the shape of the system. To extract the essence of sloshing, we derived linearized equations of fluid motion, in which the free surface remains planar (i.e., eliminating nonlinear waves imposed on the free surface). Although $\zeta$ for different shapes of stirrer is measured a priori to the operation of system, we can reasonably estimate the operating condition at which the sloshing motion becomes violent. Our oversimplified modeling may result in inconsistencies between the $\varGamma$–$\zeta$ curves. Nevertheless, the model qualitatively indicates that the sloshing becomes violent when the resonance frequency is imposed, and the damping ratio is sufficiently small. Improvement of mechanical modeling is a key subject to predict further details of fluid motion, e.g., sloshing amplitude, torque behavior, and bubble/droplet formation.

## Conclusion

We performed experiments on two-phase flow mixing in which the system comprised a rotating non-axisymmetric stirring bar in a stationary cylindrical casing. Visual observations of the gas-liquid distribution helped to classify the flow modes, which depend on the rotation speed of the stirring bar. For a sufficiently low rotation speed $\varOmega \le \mathcal {O}(10)\,\textrm{rpm}$, the flow exhibits ‘stratified flow’ owing to the gravity force acting on different densities of the gas and liquid phases; a light gas layer forms above, while a heavy liquid layer forms below. For sufficiently high rotation speed $\varOmega \ge \mathcal {O}(100)\,\textrm{rpm}$, ‘annular (concentric air in liquid) flow’ appears because the centrifugal force is dominant rather than the gravity force; the liquid phase moves along the casing wall. Between these flow patterns, a periodic surface wave is imposed owing to the interplay between the effects of gravity and inertia.

We found that sloshing becomes remarkable when the frequency of the impulsive force imposed by the stirring bar $k \varOmega$ is consistent with the natural frequency of the liquid phase $f_\text {n} (= (m g \ell /I_\text {L})^{1/2}/(2 \pi ))$. We also observed that the time-averaged torque acting on the stirring bar increased when the sloshing amplitude reached its maximum value. In a damped harmonic oscillator, energy input from an external forcing is required to maintain the oscillatory motion. For this reason, the time-averaged torque should increase when the sloshing motion becomes violent, leading to that the increase of churning loss is particularly relevant to the fluid motion, and vice versa. Finally, the maximum magnification factor of the time-averaged torque is scaled by the damping coefficient. Although the $\varGamma$–$\zeta$ curve depends on the shape of the system, for small $\zeta$, $\varGamma$ negatively correlates with $\zeta$, whereas for $\zeta \rightarrow 1$, $\varGamma$ saturates at unity. The derived equation of motion models the oscillatory motion of the free surface, which remains planar under the assumption of the simplest point-mass modeling using the Lagrange equation. The limitations of our modeling deserve further attention, such as the effect of nonlinear waves or quantification of the magnification of the time-averaged torque. This study lays the foundation for predicting violent sloshing in turbo-machineries or vehicle power trains as well as in mixing facilities.

## Figures and Tables

**Fig. 14:**
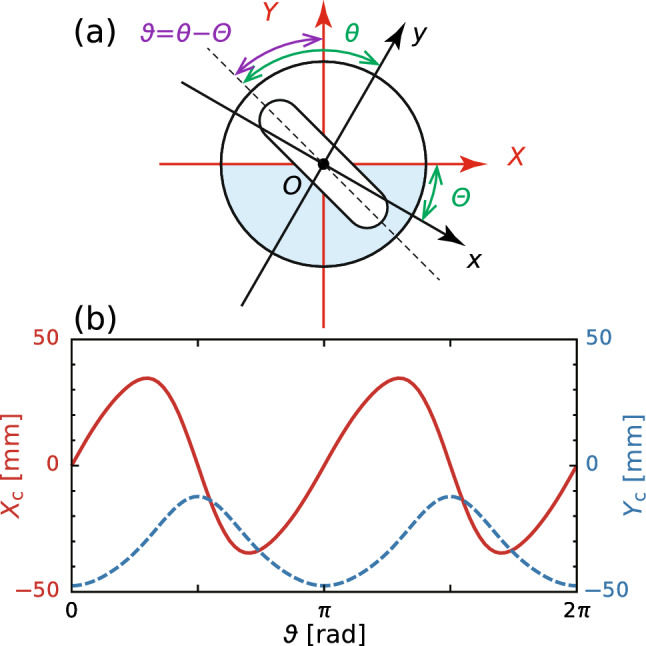
**a** Schematic outline of the stirring bar angle and liquid distribution and **b** the relationship between the center-of-gravity of the volume of stirring bar submerged in liquid and the relative the angle of bar $\vartheta$

## Data Availability

The data that support the findings of this study are available from the corresponding author upon reasonable request.

## Appendix A: Estimation of center of mass

We calculate the center of mass below the free surface. We define the virtual angle $\vartheta = \theta - \varTheta$, where $\theta$ is the angle of the stirring bar and $\varTheta$ is the angle of the free surface (see the left panel of Fig. 14). As the shape of stirring bar is given, we can numerically obtain the center of gravity $X_\text {c}$ and $Y_\text {c}$ in the virtual flame with respect to $\vartheta$ as follows:

A.1 $$\begin{aligned}&{\left\{ \begin{array}{ll} X_\text {c} (\vartheta )= -2 \displaystyle \sum _{n=1}^{\infty } n C_{2n} \sin (2n \vartheta ), \\ Y_\text {c} (\vartheta )= C_0 + \displaystyle \sum _{n=1}^{\infty } C_{2n} \cos (2n \vartheta ), \end{array}\right. } \end{aligned}$$

A.2 $$\begin{aligned}&{\left\{ \begin{array}{ll} C_0=-32.196, \\ C_2=-17.139, \\ C_4= 2.292, \\ C_6=- 0.550, \end{array}\right. } \end{aligned}$$

where $C_n$ is the coefficient of the Fourier series, where the unit is mm. $X_\text {c}$ and $Y_\text {c}$ are shown in the right panel of Fig. 14. The rotational transformation is applied to $X_\text {c}$ and $Y_\text {c}$, we then obtain the center of mass $y_\text {c} (\theta , \varTheta ) = X_\text {c} \sin \varTheta + Y_\text {c} \cos \varTheta$ in the laboratory frame as follow:

A.3 $$\begin{aligned} y_\text {c} (\theta , \varTheta )&= -2 \sum _{n=1}^{\infty } n C_{2n} \sin \left\{ 2n (\theta - \varTheta ) \right\} \sin \varTheta \nonumber \\&\quad + \sum _{n=1}^{\infty } C_{2n} \cos \left\{ 2n (\theta - \varTheta ) \right\} \cos \varTheta \nonumber \\&\quad +C_0 \cos \varTheta . \end{aligned}$$
